# Building linkages between private pharmacies and public facilities to improve diabetes and hypertension care in urban areas of Nepal: a protocol for implementation research

**DOI:** 10.1186/s13690-025-01586-4

**Published:** 2025-06-19

**Authors:** Grishu Shrestha, Deepak Joshi, Helen Elsey, Abhigyna Bhattarai, Parash Mani Sapkota, Bassey Ebenso, Raju Raman Neupane, Bryony Dawkins, Sampurna Kakchapati, Sujan Poudel, Shreeman Sharma, Abriti Arjyal, Sushil Chandra Baral

**Affiliations:** 1 HERD International, Lalitpur, Nepal; 2 Hull and York Medical School, University of York, Heslington, UK; 3 Leeds Institute of Health Sciences, University of Leeds, Leeds, UK; 4 Academic Unit of Health Economics, Leeds Institute of Health Sciences, University of Leeds, Leeds, UK

**Keywords:** Pharmacy, Diabetes, Hypertension, Public-private linkage, System-linkage, Nepal

## Abstract

**Background:**

Rapid urbanization is accelerating in low- and middle-income countries (LMICs), which impacts health behaviors and contributes to noncommunicable diseases (NCDs), such as diabetes and hypertension. As primary care services are overstretched, urban residents rely on pharmacies, creating an urgent need to implement evidence-based approaches such as the World Health Organization’s Package of Essential Non-communicable Diseases (PEN) to reach low-income households at risk of hypertension and diabetes. This study aims to identify the facilitators and barriers to the adoption, implementation, and long-term delivery of strategies to link pharmacies with public facilities in Pokhara Metropolitan City Nepal, to improve diabetes and hypertension prevention and management among poor urban populations.

**Methods and analysis:**

This study uses a sequential mixed-method design within the RE-AIM framework. Data from client surveys will assess the costs and effectiveness of system linkages and interventions in improving diabetes and hypertension screening, management, and referral. Data will be collected at four time points from at least 20 clients per pharmacy and public health facility at baseline, midline, and endline and, to assess maintenance of delivery, post endline. During each time point, repeat questionnaires will be used to assess clients’ adherence to lifestyle and referral advice. The quantitative data will be analyzed via descriptive statistics and logistic regression models to identify factors associated with change in major outcomes. Qualitative data from semi-structured interviews with health workers at pharmacies, clients, and public health facility staff will be analyzed via thematic analysis to identify barriers to and facilitators of intervention adoption, implementation, and sustainability. Endline and post-endline surveys will replicate baseline methods to evaluate intervention impact.

**Discussion:**

This study will provide insights into how private pharmacies can be linked to the public health system to provide appropriate, quality services for diabetes and hypertension within the context of a pluralistic urban health system. Using the RE-AIM framework will enable assessment across implementation domains, providing valuable insights for local governments and health systems within Nepal. Given the rapid urbanization and increasing prevalence of NCDs, which characterize the majority of LMICs, our study contributes to the understanding of how to implement such strategies to meet the needs of the urban poor in other similar contexts.

**Supplementary Information:**

The online version contains supplementary material available at 10.1186/s13690-025-01586-4.

**Table:** 

**Text box 1. Contributions to the literature**
• This protocol focuses on the integration of private pharmacies with public health institutions within the context of the management of diabetes and hypertension in understudied urban informal settings.
• It addresses a key gap in understanding healthcare services in rapidly urbanizing areas with urban poor populations.
• This may help develop reproducible interventions that can enhance hypertension and diabetes management in healthcare systems in low- and middle-income countries, where these two diseases are becoming a public health crisis.

## Background

More than half of the world’s population now lives in urban areas, and urbanization is occurring rapidly in most low- and middle-income countries (LMICs), especially in Asia and Africa [1]. Although urban areas have access to better health and other services that promote health, such as improved household energy, water, and sanitation, there are also increased health risks, such as a lack of physical activity, low-quality food products, and poor air quality. The combination of these risks is fueling the rise of non-communicable diseases (NCDs), such as diabetes and hypertension [2]. According to the World Health Organization (WHO), 77% of all NCD deaths occur in LMICs [3], and these deaths are predicted to increase by more than 50% in LMICs by 2030 [4].

This pattern of rapid urbanization and an increase in the prevalence of diabetes and hypertension is clearly observed in Nepal, which has the fastest urbanization rate in South Asia [5]. The recent WHO STEPwise approach to NCD risk factor surveillance (STEPS) survey (2019), which follows a standardized method to assess the prevalence of NCDs and their associated risk factors, reported that the prevalence of diabetes and hypertension among adults aged 15–49 years were greater in males, with the prevalence of diabetes being 6.3% in males and 5.3% in females, whereas the prevalence of hypertension was 29.8% in males and 19.7% in females. Notable disparities in the prevalence of diabetes and hypertension were found in Nepal, with urban areas exhibiting higher rates than rural regions; for example, the prevalence of hypertension was higher in urban areas (26.5%) than in rural areas (23.8%), and similarly, the diabetes prevalence was 7.2% in urban areas, whereas it was 4.6% in rural regions in the Kathmandu Valley and high urban growth in Pokhara Valley, the Inner Terai Valleys, and border towns [6]. With expanding municipal areas, slums or slum-like settlements are also expanding rapidly. According to World Bank data from 2020, an estimated 40% of the population of Nepal lives in slum settlements [7]. Owing to the double burden of NCDs and communicable diseases, coupled with a growing urban population, healthcare systems in LMIC cities are not well equipped to respond to these multiple challenges [8, 9].

With public primary care services overstretched and limited availability, most urban residents use private facilities and pharmacies. Considering this, pharmacies have been identified as an untapped resource for increasing access to primary care among the poor in urban areas [10] and preventing chronic conditions [11]. However, a systematic review of pharmacy performance in Asia highlighted issues such as a lack of appropriate referrals, poor medical history, inappropriate prescribing, and limited counseling [12]. Despite these challenges, evidence shows that pharmacists can effectively improve blood pressure control [13] and control HbA1c [14]. While high-income countries have integrated pharmacies into primary care, there is limited evidence from LMICs, including Nepal, on how to do this effectively for managing hypertension and diabetes [15].

The WHO has developed a package of essential noncommunicable disease interventions (PENs) to manage the increasing number of NCD cases in low-resource primary care settings [15]. The PEN package includes diagnosing NCDs, addressing risk factors, early detection, health education and treatment plans, referrals, and follow-up [16]. Nepal has adopted the PEN as part of its multisectoral action plan for NCDS [17] at primary healthcare facilities nationwide. Providing knowledge and skills to deliver PEN is just one challenge; systems are also needed for supervision, quality monitoring, referrals, and record keeping. In Nepal’s federalized system, the metropolitan city (local government) is responsible for providing services such as PEN implementation, supervision, referral, and reporting as part of its basic health services package [18].

HERD International (HERDi)’s partnership with Pokhara Metropolitan City (PMC) now stands for more than seven years, building on trusted collaboration in urban health systems to develop evidence for private-sector partnerships. Together with PMC, HERDi has co-created the intervention through needs assessment, prioritization, and co-implementation and evaluation. In 2022, a need assessment at Pokhara revealed barriers to diabetes and hypertension care, particularly in low-income areas [19]. These included low-risk perceptions toward NCDs, poor treatment compliance, misconceptions about medication for hypertension and diabetes, and perceptions of poor quality at public facilities. Poor communities often choose local pharmacies for healthcare because of proximity, promptness, and trust [19]. With the objective of providing information and identifying key issues workshops were conducted with different stakeholders. The workshop mainly focused on identifying key issues and possible solutions to it through cause and effect model. Based on the identified issues and solutions, prioritization workshop was held with the stakeholders. The prioritization was done based on availability of resources, capacity, severity of the issue, and priority of the PMC health system, where diabetes and hypertension were identified as major health issue of the city and role of private sector is must to address the issue, particularly the pharmacies who were offering services as first point of contact. The co-creation process involved stakeholders in developing training, materials, and supervision for community pharmacies that are private owned, to provide screening, advice, and referrals for diabetes and hypertension in line with the PEN package while also strengthening the management of these conditions in primary care facilities through formal referrals.

## Objectives

This paper presents a protocol for an implementation research study to evaluate strategies to link private pharmacies with public facilities to improve diabetes and hypertension care in line with national guidelines based on the WHO PEN package. The study aims to understand the extent to which the implementation strategies will ensure that the intervention reaches those with or at risk of hypertension and/or diabetes from low-income urban households and the facilitators and barriers to the adoption, implementation, and sustainable delivery of the intervention within Pokhara Metropolitan City (PMC), Nepal.

## Methods and design

### Study design

This implementation research uses a sequential explanatory mixed-method approach [20] with an uncontrolled before-and-after design. The RE-AIM framework [21] will be used to structure the study to identify the extent to which the intervention reaches the urban poor; can improve screening, advice, and referrals for diabetes and hypertension care; and is adopted by pharmacies. We will also identify any facilitators or barriers to the implementation and sustainability of the intervention.

## Selection of study sites

As part of our codesign workshop, and in collaboration with PMC- Health Division (PMC-HD), we identified the study site as the catchment area of basic 15-bed hospitals implemented under the local government. The area consists of five of the 33 administrative wards in the PMC and includes 6 public primary health care facilities and ~ 30 pharmacies, of which 11 pharmacies were included in this study, as they currently provide diabetes and hypertension care services, are registered with the PMC-HD, are available as paramedics, and expressed a willingness to participate. Paramedics are the ones with diploma course like nurses, health assistants, auxiliary health workers.

## Implementation strategies

**Fig. 1:**
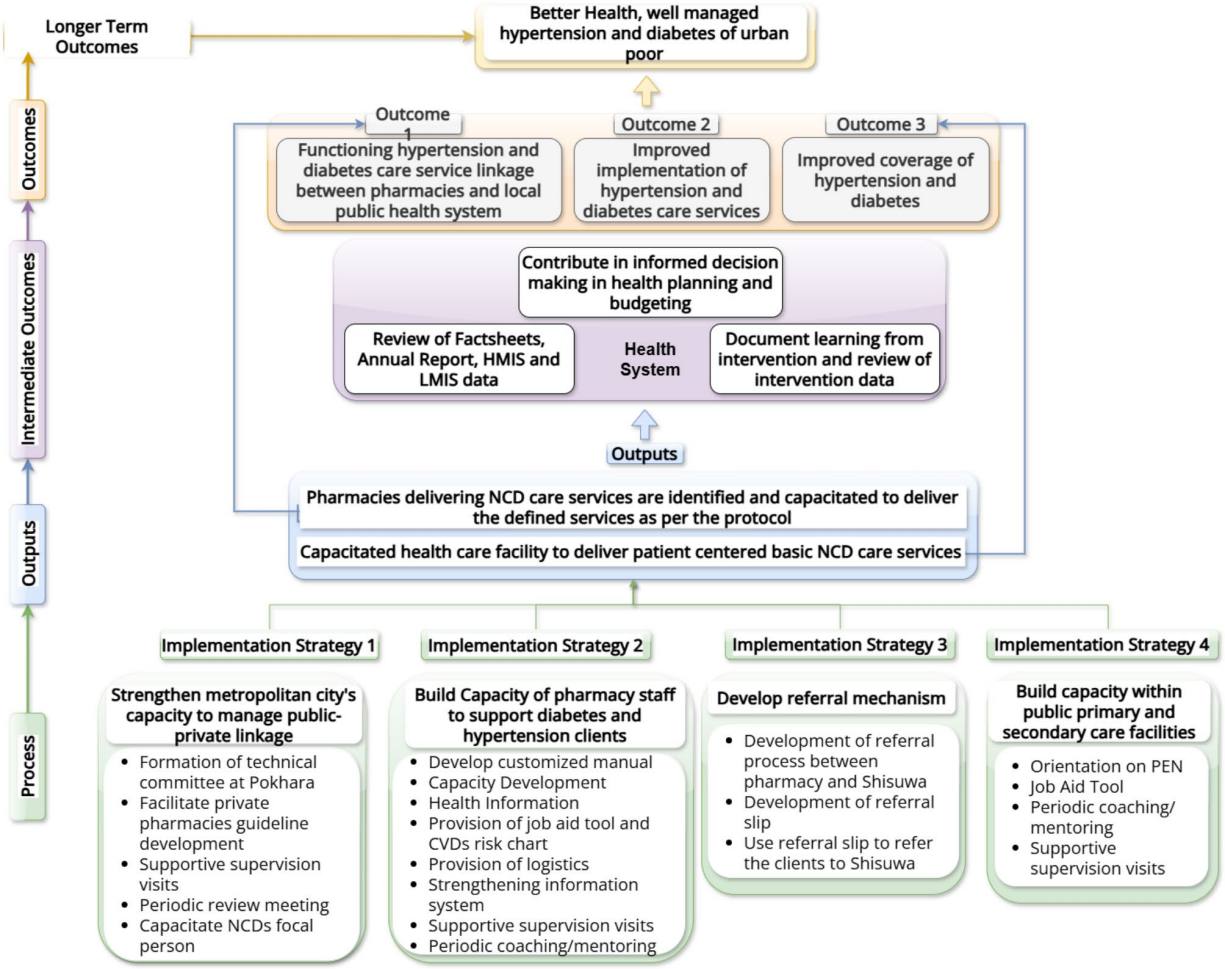
Theory of change

To integrate pharmacies into the public health system to deliver improved access to diabetes and hypertension prevention and management, the codesign process identified four key implementation strategies: (i) strengthening the PMC health division (PMC-HD) to manage linkages, (ii) improving the knowledge and practices of health workers at pharmacies to prevent and support management of hypertension and diabetes, (iii) establishing an effective referral mechanism between pharmacies and higher-level public and private facilities, and (iv) ensuring that pharmacy clients referred to public facilities receive appropriate care, strengthening the capacity of public facilities to deliver hypertension and diabetes care. The combination of these implementation strategies is expected to result in improved coverage, implementation, and service linkages to ultimately ensure that the urban poor have well-managed hypertension and diabetes for good health (see Fig. 1 below).

## Theory of change

Details of the interventions within each of the four implementation strategies are provided below:

**Implementation strategy 1**: Strengthen PMC-HD's capacity to manage public-private links. this strategy is subcategorized into five strategies:

- **Formation of the technical committee at the PMC Health Division**: A committee chaired by the PMC-HD chief with members, including focal personnel of NCDs and health management and information systems, one doctor and one nursing officer from a tertiary hospital (Pokhara Academy of Health Science-PAoHS) will be formed and facilitated by HERDi. The committee will be responsible for guiding the intervention implementation, particularly in reviewing and endorsing the customized PEN package for pharmacies.
- **Facilitate pharmacy guideline development**: We will assist in creating guidelines for private pharmacies to ensure that they align with the overall NCD management strategy. These guidelines will help standardize the role of private pharmacies in screening, referring, and managing clients with NCDs, promoting a more coordinated approach across all health workers.
- **Supportive supervision visits**: These visits will involve personnel from the technical committee, including experts in the PEN package and stakeholders from PMC-HD. They will provide guidance, monitor progress, and address any challenges in implementing NCD interventions. Supervision visits will be conducted by the members of the technical committee at an interval of three months with support from HERDi.
- **Periodic review meetings**: In every quarter, there will be periodic review meetings with health workers of pharmacy and public healthcare providers (i.e., 11 pharmacies, 6 public health facilities and 1 referral hospital) along with technical committee team members. This meeting will cover the review of data from record registers, periodic visits, supervision checklists and reflections from intervention work. HERDi will analyze the data, identify patterns or issues, and develop ideas for improvement. The meeting will also include discussion regarding the need to revise the IEC materials (brochure). This process helps ensure ongoing improvement in service quality.
- **Capacitate the NCD focal person**: We will enhance the skills and knowledge of the designated NCD focal person at each health facility. This includes providing PEN package training to ensure that they are well equipped to lead NCD management efforts effectively.

**Implementation strategy 2**: Build the capacity of health workers at pharmacy to support diabetes and hypertension patients in the following 8 steps:

- **Develop customized manual**: One manual has already been developed by the Government of Nepal, an adapted version of the WHO PEN. The guidelines will be revised and adapted for use in pharmacies to draw on content related to diabetes and hypertension. The service areas within the content will include screening, advice, referral, recording and reporting.
- **Capacity development**: Orientations of customized PEN protocols, including health information materials, will be provided to pharmacies (health worker at pharmacies) on screening, advice, referrals, records and reports. To screen for hypertension and diabetes, all people visiting pharmacies for any service will be screened for their age (i.e., 40 years and above). The health workers of pharmacy will receive training related to the screening process of the clients.
- **Health information**: Customized brochures for clients to cover health promotion messages, risk factors and symptoms of diabetes and hypertension will be provided. The messages will be guided by basic health service guidelines^1^.
- **Provision of job aid tool and CVD risk chart**: A handy chart and step-by-step guide based on the PEN protocol will be given to health care providers of the pharmacy (Supplementary File 2). This tool will help them manage NCDs at the pharmacy. A flipbook with a calendar that contains health messages for the provider at the pharmacy to use as reference material during consultations will also be provided. A CVD risk chart will also be provided to pharmacies so that they can quickly categorize the client’s risk of developing CVD in the future.
- **Provision of logistics**: We will provide necessary items such as weighing machines, height measurement tools, CVD risk charts, blood pressure monitors, and glucometers to all pharmacies.
- **Strengthening the information system**: We will implement a recording form to institutionalize the information linkage and support informed decision making to improve the NCD care service linkage. The government-developed record register for NCDs (HMIS 5.9) will be adopted to record the client’s data (Supplementary File 3). The orientation will also include a session on the recording tool and exercises. HERDi will collects information from the record register maintained at the pharmacy at the end of each month. The cumulative data are then entered into HMIS because the PMC health division will provide a separate username and password for logging into the system.
- **Supportive Supervision Visits**: Supportive supervision will be conducted in pharmacies as mentioned above in implementation strategy 1.
- **Periodic coaching/mentoring**: Biweekly periodic coaching and mentoring for the first three months followed by a one-month visit will be provided by the HERDi team. The HERDi team will offer advice, help solve problems and share best practices to keep providers adhering to the protocol in managing and recording the cases of NCDs.

**Implementation strategy 3**: Develop an effective referral mechanism as follows:

- **Development of a referral process between pharmacies and Shisuwa Hospital**: Despite pharmacies screening clients and identifying them as having the disease/condition or as being at risk of the disease/condition, there might be a lack of uniformity in referral and management. Through this intervention, we aim to systematize a way forward to specify how referred patients will be recorded in the referral slip (Supplementary File 4). This referral process will be customized as a part of this intervention.
- **Development of a referral slip**: There will be three slips (2 of which are carbon copies) per referred client. One original slip will be provided to the client, another (one copy) will be provided by pharmacies, and the last copy will be reported to public health facilities.
- **A referral slip is used to refer clients to Shisuwa Hospital**: The pharmacy will use the referral slip while referring the clients to Shisuwa Hospital. The referral slip will have a section where the hospital needs to fill out the return information and send it with the clients to the health facility from where they were referred. Since one of the copies of the referral slip will be kept in pharmacies, researchers will visit monthly to collect data from the register and all the referral slips. If referred by a pharmacist, the client will be asked to give the original referral slip to the provider that they visit. The research team will visit the referral center to collect the referral slips from there as well.

**Implementation strategy 4**: Build capacity within public primary and secondary care facilities

We will initiate the intervention in the public health facility with one-day refresher training on PEN for all healthcare providers in the selected public health facility. Prior to the refresher training, an assessment will be conducted to evaluate the readiness of these facilities for NCD care services. This will be followed by the following four activities:

- **Orientation of the PEN**: All healthcare providers (Health Facility In charge- Auxiliary Health Worker or Health Assistant) will attend training sessions to learn about the PEN guidelines. These sessions will explain how to use the job aid tool and how to apply the guidelines in their daily work, helping them feel confident in delivering PEN services.
- **Job Aid Tool**: A calendar flipchart will be provided to the facilities so that they can deliver health messages uniformly across the facilities.
- **Periodic Coaching/Mentoring**: Healthcare providers will receive regular coaching and mentoring from the HERDi team, as mentioned in implementation strategy 2.
- **Supportive Supervision Visits**: Supportive supervision will be conducted in public facilities as mentioned above in implementation strategy 1.

## Data collection measures and processes

The study is structured into four phases of data collection: baseline (before intervention), midline (month 6), endline (month 12) and post-endline (6 months after the end of implementation). Data collection will include the following measures:

### Client questionnaire survey

At baseline, we will approach and obtain consent from 11 private pharmacies, 6 primary healthcare facilities, and one basic hospital. Each pharmacy and public facility, with the client’s consent, will record the contact details of the clients visiting the facility after they agreed to participate in the study. Our team of 16 trained field researchers will then approach the clients initially by phone and will visit their homes for data collection following their consent. We aim to recruit at least 20 clients from each facility (all the 11 pharmacies, 6 public health facility and hospital) over a period of 15 days. This equates to 360 clients who will complete a questionnaire to assess self-reported diabetes, hypertension, and risk factors (smoking, alcohol, exercise, diet–salt), health-seeking behavior, their experience of screening, referrals, advice/counseling from the provider visited, and economic costs (opportunity cost, treatment cost, transport) of the last visit and demographic details. These same 360 clients will be interviewed 2 weeks later using a different questionnaire to identify any changes they have made to their lifestyle behaviors, management of their diabetes and/or hypertension, any visits to the referral site, and treatments given.

The questionnaires will be pretested with a similar peri-urban setting in Lalitpur. Data collection will be done from different clients at each phase. But at each phase data will be collected from the same clients at two point of time. Respondents recruited at baseline, midline, endline and post-endline are independent to each other. However due to relatively small geographic setting, the clients recorded at pharmacies and health facilities can be repeat clients, and hence there is chance of them being selected in multiple survey points. Midline data collection will occur at 6 months of intervention, with end-line surveys after 12 months of intervention implementation following the same methods and tools as those used at baseline. This process will also be repeated 6 months after the end-line. We expect each phase of data collection to take 2 months.

### Health facility assessment (pharmacy and public health facility)

We will collect quantitative data from all the 11 pharmacies and 6 public primary health care facilities in each phase. The questionnaire will include the readiness assessment (availability of logistics and drugs), information of human resource (number of staffs, their designation, experience, training on PEN), and information system.

### Qualitative interviews with healthcare providers and system actors

We will conduct interviews with six public health care providers (one staff per public health facility) regarding their perceptions of the role of pharmacies in delivering NCD services, the implementation of NCD services, and any unintended consequences of the pharmacy linkage. We will purposively sample system actors to conduct key informant interviews to understand any wider system issues arising from implementation, if the intervention reaches certain sectors of poor urban communities and explore the potential for the sustainability and scale-up of interventions. The interviews will be conducted at four time points, i.e., baseline, midline, endline, and six months post-endline.

### Qualitative interviews with health workers at pharmacy

We aim to interview at least one staff member from each of the 11 pharmacies at all four points in time. Within the 11 pharmacies, if there are other pharmacy personnel who are delivering services, then they will also be interviewed. We will purposefully sample the health workers providers at pharmacy on the basis of their motivation to deliver the intervention to explore reasons for adoption/non-adoption and explore their experiences, challenges, facilitators, any unintended consequences (such as changes in prescribing) and the extent of fidelity during delivery of the intervention.

### Qualitative interviews with clients

At each of the specified time points of the implementation phase, we will conduct 20 interviews with clients who will be purposively selected on the basis of diversity of age, gender, occupation, and location (slum/non-slum). Qualitative interviews with clients will help to understand their experience with the intervention and their experience with the services, which are based on the type of service providers at the pharmacy. We will also include 5 clients who have been referred from the pharmacy to the referral hospital to understand their experiences with the services received and referrals. Interviews with the referral client will be collected at the specified time points throughout the implementation and post-end line periods.

### Routine data

Individual patient data will be collected every month during the intervention period from the Health Management Information System (HMIS) routine data. We will create our own database of individual patients (anonymized), adding data to their case files over the one-year time frame of the intervention. If complete, the HMIS data will include details of age, sex, ethnicity and location.

### Observation visits

The research team will make regular observations of pharmacy practices (at least once per month) at each pharmacy. These visits will last approximately 45 min with each pharmacy during the one-year implementation phase. If the pharmacy is too busy or closed, the team will return at a more suitable time to conduct the observation. While at the pharmacy, the researcher will discuss the implementation of the intervention with the health workers at pharmacy, and if a client over 40 years visits the pharmacy during our visit, the researcher will observe the interaction between the health workers at pharmacy and the client. A checklist will be used to record which staff member is working in the pharmacy during the visit; which components of the intervention are visible and/or actively being used by the health workers at pharmacy; and if an eligible client (i.e., over 40 years) visits, the appropriateness of the screening, advice or management/referral services provided for the hypertension or diabetes will be observed.

### Implementation framework

To understand the facilitators and barriers to the implementation, reach and sustainability of the intervention, we will use the RE-AIM framework of reach (R) of the intervention to the target population of 40 years or older with or without diabetes and/or hypertension from low-income urban families; the effectiveness (E) of the intervention in improving the screening, referral and counseling of clients by health workers at pharmacies and improvements in client self-care and satisfaction with services; the extent to which the intervention is adopted (A) by public health facilities, different types of pharmacies and health workers at pharmacy; and the fidelity to the intervention, facilitators, barriers to, the costs and any unintended consequences of implementation (I) and the maintenance (M) of implementation six months after the end of the one-year study implementation period. RE-AIM has been used extensively in public health interventions over the last two decades and provides a useful structure to inform future implementation and scale-up of interventions [21].

Our research questions for each component of the RE-AIM evaluation framework are as follows:

#### Reach

What proportion of the poor urban population aged 40 years and older participate in screening, lifestyle advice, and referrals for diabetes and hypertension? What proportion is referred and what proportion actually goes for follow up?

#### Effectiveness

Does the public-private linkage system improve screening, referrals and advice for hypertension and diabetes patients as per the PEN? Does this system improve client satisfaction and management of diabetes and/or hypertension?

#### Adoption

What proportion of private pharmacies and their staff adopt the linkage system [information linkage, service linkage, i.e., screening, counseling, referral]?

What are the characteristics of the adopters?

What are the characteristics of pharmacy settings and staff that adopt the linkage system, and which factors encourage or discourage adoption?

#### Implementation

What are the facilitators, barriers, and costs of public-private linkage operating system in Pokhara?

How does the linkage system implemented in practice compare to the initial design (fidelity), and what adaptations are made?

Are there any unintended consequences for pharmacies, clients, or the public health system?

#### Maintenance

How does the implementation of public-private linkage systems change over the one-year implementation period of the intervention, and which components of the system are still being implemented 6 months after the study period?

The RE-AIM framework acts as an overall structure for our qualitative analysis. By following the framework approach [22], we inductively generate codes from our data organized under the 5 domains of the RE-AIM framework. The quantitative and qualitative findings are compared within a meta-inference table to understand the complexities of implementation from multiple perspectives [20].

#### Reach

Our primary target population is urban poor individuals, specifically those 40 years old and above, who are at risk of or already hypertensive or diabetic. As a proxy for urban poverty, we will use location (slum/non-slum area) and main income source (daily wage, petty business, social security). We wish to understand the extent to which clients receiving the linkage intervention differ by occupation, gender, ethnicity, and age, captured through questionnaires. We will explore these characteristics (gender, age, ethnicity) of our participants with wider Pokhara census data to identify any excluded groups. On the basis of our quantitative analysis of the questionnaire data at baseline and midline time points, we will purposively select participants to explore differing uses of pharmacies and primary care among poor urban men and women with different occupations, ethnicities, locations (slum/non-slum areas), main sources of income (particularly daily wages, skilled laborers and petty businesses, which are associated with poverty in Pokhara) and disabilities. We will use an in-depth interview guide to explore why the intervention may or may not reach certain sectors of poor urban communities. We will also analyze HMIS data to explore inequities in the reach of the intervention.

#### Effectiveness

To understand the extent to which the public-pharmacy linkage has been effective, our main outcome will be the appropriate management of pharmacy clients as per the PEN algorithm. Clients will be considered appropriately managed if they were: (i) offered screening, (ii) provided lifestyle advice and (iii) referred appropriately as per the algorithm (Supplementary File 2). Data will be collected from the recording register (i.e., data on height, weight, blood pressure, blood glucose, health information and referrals) or from the client questionnaire survey if the register is incomplete. This will be complemented by regular observations of pharmacy practices by the research team using a checklist to reflect on the accuracy of the recording register.

At the client level, we will assess the adoption of lifestyle advice, referrals, client satisfaction with services from the pharmacy and any higher-level services used. Analysis of the HMIS data will help explore changes in hypertension and/or diabetes management.

We will also combine effectiveness data with data collected on costs to inform estimates of the cost-effectiveness of the interventions from the client/health system perspective. Details of the cost evaluation will be specified in a separate cost analysis plan prior to any analysis being undertaken. Qualitative interviews will address the intervention’s impact on health and well-being.

#### Adoption

We will explore adoption among pharmacies and their staff members. We will record the number of pharmacies and pharmacy personnel invited to the training and the proportion that participated. During observation visits, we will note any pharmacies not using components of the intervention.

Each staff member will receive a unique ID, allowing us to track any staff member who is not adopting the intervention. After 6 months, we will conduct approximately 3 interviews with health workers at pharmacy who had not adopted the intervention to understand the reason for the lack of adoption and 7 interviews with those who had to explore their motivation. Within these 10 health workers at pharmacies to be interviewed, we will ensure that there is a spread across gender, location and level of training.

#### Implementation

The methods planned to assess adoption, i.e., (i) recording registers and (ii) referral slips (as above), will also provide detailed information on the facilitators and barriers to the implementation of screening, health information, reporting by pharmacies and referral patterns between pharmacies and public health facilities. We will assess healthcare providers’ knowledge before and after they are trained on the PEN package. The regular observation visits will enable the identification of the pharmacy that is serving the clients, the utilization of intervention materials and the experience of using the intervention materials. An observation checklist will be completed for eligible clients, ensuring pharmacy compliance with intervention processes.

From the supportive supervision visits, we will review the supervision checklists (Supplementary File 1) completed by supervisors from PMC, who will be interviewed to understand their experiences in providing supportive supervision. We will also interview key PMC staff, including public health inspectors, who are engaged in supervision visits to understand changes in policy and guidance on working with pharmacies over the study period. This will allow us to identify any impact of revised guidance or changes within PMCs on intervention implementation.

Learning and revising the implementation strategies: Periodic review meetings will be used to review the changes and, where relevant (within time and resources), adapt the intervention components. The periodic review meetings will be audio-recorded and transcribed, contributing to the qualitative data.

#### Costs

We will assess the costs of the public‒private linkage intervention from both the health system perspective and the client perspective. For clients, we will estimate direct medical (e.g., medication, tests), nonmedical (e.g., transportation) and indirect costs (e.g., lost productivity). The productive time lost will be converted to a monetary value via a human capital approach [23] by applying actual wages in Nepal [24]. For the health system, we will use a bottom-up approach to estimate costs per client, including recurrent costs such as training, materials, and any time spent delivering consultations/counseling. Time costs will be converted to monetary values on the basis of the salary of each professional category and the position in Nepal [25]. Costs will be reported in Nepalese Rupees and converted to US dollars via an average exchange rate from OANDA [26].

During the last six months of the implementation period, interim analysis will be conducted by triangulating the interviews, periodic visits, referral slips and reporting forms to understand the extent to which a component of the intervention has been implemented. This will help identify pharmacies with high fidelity to the intervention and low fidelity.

#### Maintenance

We will assess changes in the extent of delivery of the intervention by analyzing recording registers and referral slips over a one-year implementation period and a six-month maintenance period. Client and pharmacy interviews, along with routine HMIS data on hypertensive and diabetic patients, will be analyzed to track changes in implementation and management outcomes over time. A final round of client and pharmacy interviews in the maintenance period will assess which elements of the intervention are still in place six months after the end of the implementation period. Qualitative interviews with health workers at pharmacies, PMC staff, provincial-level staff and public healthcare providers will explore their views on sustainability and the possibility of scaling up the intervention.

### Analysis

Quantitative data from the client’s surveys will be collected via an Open Data Kit (ODK) on tablets and mobile devices. The ODK forms will be encoded into Excel spreadsheets with questions and answer options available in English and Nepali. Each respondent and field researcher will have a unique ID number encoded to a QR code to link data across time points. Synchronized data from the tablets will be downloaded from cloud servers every day, anonymized, labeled, and recoded via scripts (R scripts) files to create data files for tracking progress and checking the data quality as part of the real-time data monitoring process. For data cleaning, verification and analysis, files will be downloaded in Excel and imported into R.

The data and value labels will be imported into R for data cleaning, management and analysis. In the data analysis, exploration of the data will be performed through the use of both descriptive and inferential statistics wherever relevant. Descriptive statistics will be performed to summarize the data at baseline, and repeated-measures ANOVA will be used to compare changes in blood sugar and pressure levels over the four survey time points. Further, we will perform logistic regression for the lifestyle modifications: physical activity, alcohol consumption, smoking, and salt intake. The model will be adjusted for key socio-demographic variables: Age, sex, education and wealth index. The model will also be adjusted for clustering of data within each pharmacy and public health facility. We will use R version 4.4.1 and RStudio 2024.4.2.764 for statistical analysis.

## Discussion

This paper outlines a mixed-method protocol aimed at building linkages between private pharmacies and public facilities to improve diabetes and hypertension care in an urban city of Nepal, particularly in the PMC. This study is the first in Nepal, to utilize a one-year intervention to assess changes over time in both public and private health facilities to manage these conditions. It focuses particularly on the process of screening, counseling, recording, referring, and reporting. As a part of an intervention, we will provide training to private pharmacies on customized PEN protocols, along with job aid tools, IEC materials, periodic coaching, and supervision. These changes will be further assessed via the RE-AIM framework to report the process and progress with adoption and implementation throughout the intervention period as well as the maintenance of the intervention 6 months after the project ends.

While policies emphasize public-private partnerships, the diversity of private providers, including pharmacies and different tiers of government, has complicated attempts to develop guidelines with a specific modality of partnership with clarity on how pharmacists should provide appropriate services. This study aims to generate evidence to support local governments in developing strategies to facilitate effective engagement with private pharmacies in providing quality healthcare services that reach the urban poor. Given the rapid growth of urban areas and the strain on public healthcare services, particularly for NCDs, this evidence is urgently needed.

This study comprehensively assesses the opportunity to maintain public-private partnerships with PMCs and their sustainability within the health system. This study is valuable for those interested in applying similar methods for assessing health system interventions and offers insights for policymakers and practitioners.

### Strengths and limitations

While our study is limited to focusing on only 11 private pharmacies and six public health facilities in the PMC, we will collect both quantitative and qualitative data from healthcare providers, health workers at pharmacies, and clients. The mixed-methods approach is a key strength and is aligned with the RE-AIM framework for an in-depth exploration of implementation. Although a larger sample of pharmacies and public health facilities would have been beneficial, it would have been unfeasible within the study resources, and a comparator would offer limited value owing to the lack of formal referrals processed in pharmacies.

## Electronic supplementary material

Below is the link to the electronic supplementary material.

Supplementary Material 1

Supplementary Material 2

Supplementary Material 3

Supplementary Material 4

Supplementary Material 5

Supplementary Material 6

Supplementary Material 7

Supplementary Material 8

## Data Availability

No datasets were generated or analysed during the current study.

## Footnotes

1 Ministry of Health and Population; Basic Health Services Standard Treatment Protocol, 2078.
