# Supplementary material for: Building linkages between private pharmacies and public facilities to improve diabetes and hypertension care in urban areas of Nepal: a protocol for implementation research
Source: Arch Public Health. 2025 Jun 19;83:160. doi: 10.1186/s13690-025-01586-4 (PMC12178029; doi:10.1186/s13690-025-01586-4)
Supplement: Supplementary file 1 — Supplementary Material 1 [file 13690_2025_1586_MOESM1_ESM.pdf]

Checklist for supportive supervision and monitoring

Supervision date: From…………to………..… Name of HF:

Name of the supervisor: ……………………………………………………………..

S.N.	Supervision Areas	Observation of the supervisor<br>Y=Yes, N=NO, DN=Do not Know, ST=Some time	Actions agreed by the HW and supervisor for improvements
1	**Administration and management**		
1.1	Are the quality improvement teams formed?		
1.2	Describe whether quality enhancement discussions are conducted monthly. (Review reports of the meetings)		
2	**Logistics (medicines, supplies & equipment)**		
2.1	Are the essential medicines required for the treatment of hypertension available (in stock) to last for 2 months		
2.2	Are the essential medicines required for the treatment of Diabetes available (in stock) to last for 2 months		
2.3	Is treatment protocol available there?		
2	Is the blood glucometer in use?		

4	Is the BP set working		
2.5	Is a weighing scale available?		
2.6	Is measuring tape available		
3	**Record keeping**		
3.1	Is the register updated?		
3.2	Is patient registration documented and well organized?		
3.3	Is the NCD monthly reporting timely		
4	**Patient care**		
4.1	Can health service providers recall the purpose of the CVD risk prediction chart?		
4.2	Is a CVD risk score routinely provided to eligible patients?		
4.3	Is there proper risk factor counseling for patients		
4.4	Is there separate responsible HR available (Only for HF)		
4.5	Is there an established follow-up mechanism for patients		
5	**Integration of NCD services**		
5.1	Are service providers aware of the purpose of the PEN program?		
5.2	Is health education on tobacco, alcohol, unhealthy diet, and physical activity provided		
5.3	Are referrals occurring		
6	**Overall observations and summary**		

6.1	What would you rate the level of overall performance of NCD management on a scale of 1 to 10, 1 being minimal and 10 being excellent?
6.1.1	Rating:………….. Brief statement on the reasons for the level of rating:
6.2	Clearly, outline a summary of recommendations between the supervisor and the supervisee within an agreed set time for the next supervision
6.2.1	Summary of recommendations:

Signature: Date
