# Supplementary figures and images for: Building linkages between private pharmacies and public facilities to improve diabetes and hypertension care in urban areas of Nepal: a protocol for implementation research
Source: Arch Public Health. 2025 Jun 19;83:160. doi: 10.1186/s13690-025-01586-4 (PMC12178029; doi:10.1186/s13690-025-01586-4)

## Algorithm for Screening of Clients Visiting Pharmacy

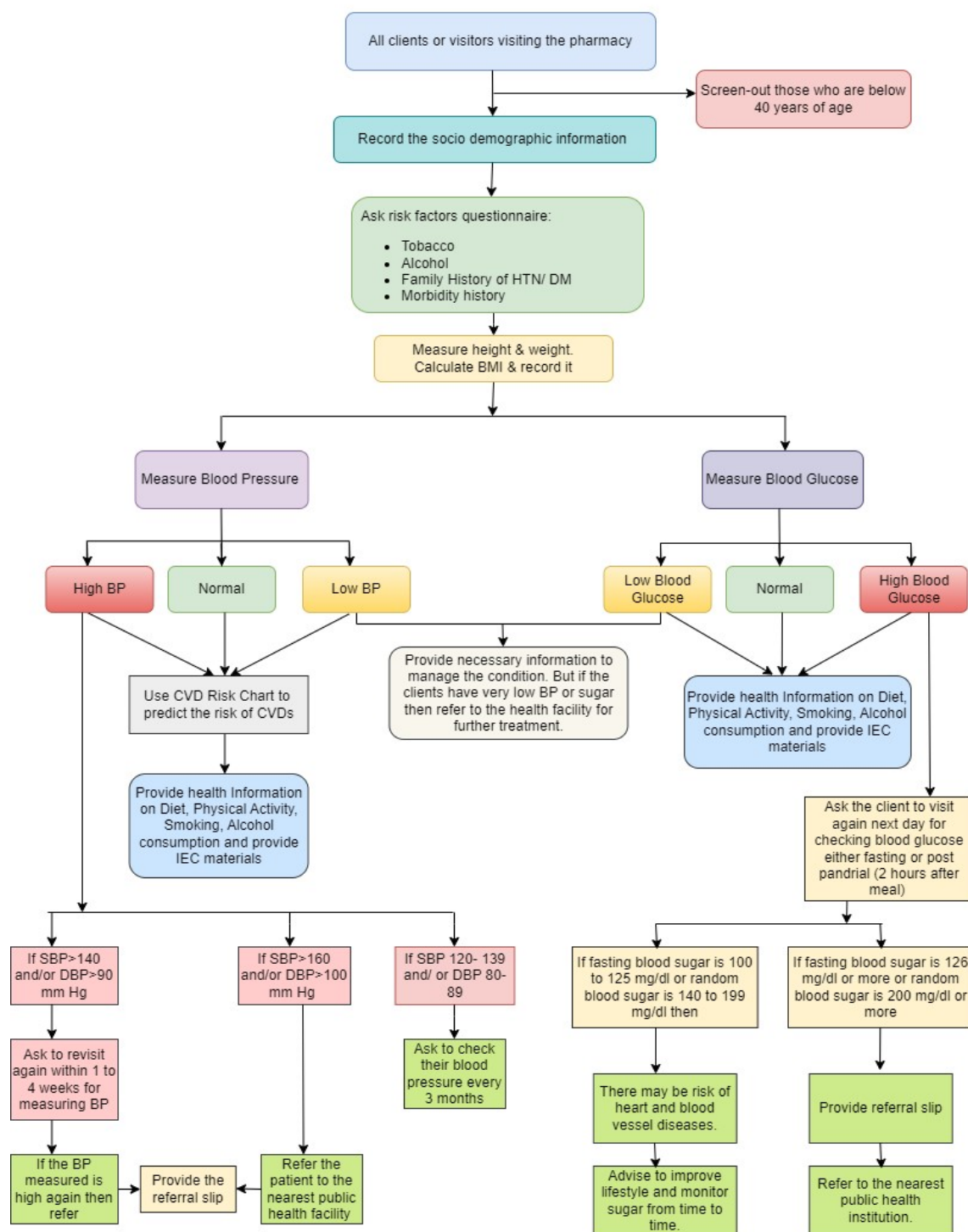

Supplement: Supplementary file 2 — Supplementary Material 2 [file 13690_2025_1586_MOESM2_ESM.pdf]

**Figure showing the process of client survey**

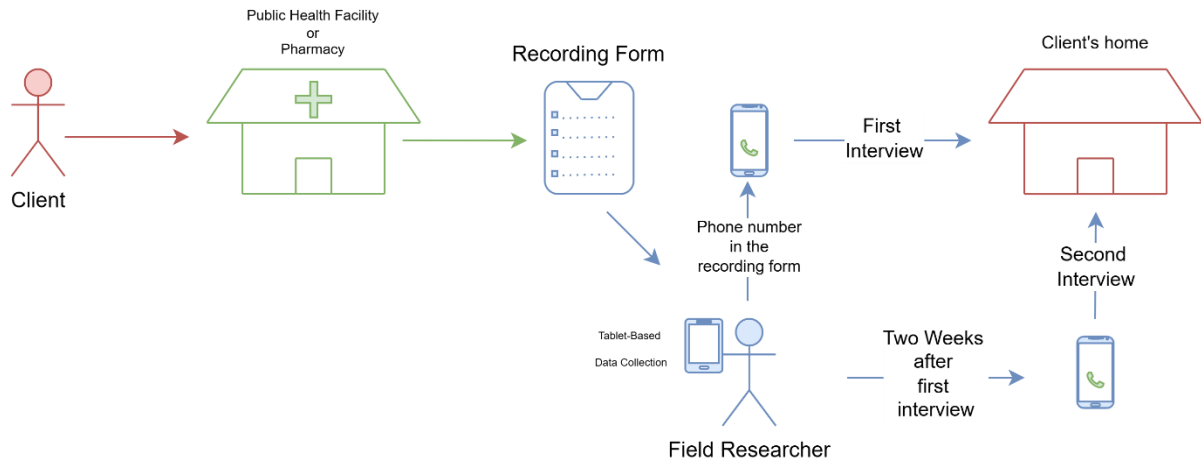

Supplement: Supplementary file 6 — Supplementary Material 6 [file 13690_2025_1586_MOESM6_ESM.pdf]
