# Supplementary material for: Building linkages between private pharmacies and public facilities to improve diabetes and hypertension care in urban areas of Nepal: a protocol for implementation research
Source: Arch Public Health. 2025 Jun 19;83:160. doi: 10.1186/s13690-025-01586-4 (PMC12178029; doi:10.1186/s13690-025-01586-4)
Supplement: Supplementary file 3 — Supplementary Material 3 [file 13690_2025_1586_MOESM3_ESM.pdf]

नसर्ने रोग तथा मानसिक स्वास्थ्य सेवा रजिष्टर

	सेवा सम्बन्धि जानकारी				सेवाग्राहीको व्यक्तिगत जानकारी					ठेगाना/ सम्पर्क नं.		निदान (Diagnosis)		सेवाको प्रकार					पुराना सेवाग्राहीहरुको लागि मात्र		प्रेषण
क्र.स.	मूल दर्ता नम्बर / अन्य सेवा दर्ता नम्बर	मिति	NCD/MH/ Injury	नयाँ/ पुरानो / सेवा दर्ता नम्बर	सेवाग्राहीको नाम/थर	जाति कोड	उमेर	लिङ्ग: महिला	पुरुष	जिल्ला / गा.पा/न.पा, वडा नं / टोल / सम्पर्क नम्बर	Investigation	Diagnosis	ICD Code	औषधि	मनोसामाजिक परामर्श	परामर्श	अन्य	मृत्यु भएको	उपचार नियमितता (Rx compliance)	सुधारको अवस्था	प्रेषण भइ आएको संस्था/ व्यक्तिको नाम / प्रेषण गरिएको संस्थाको नाम
														औषधि, उपचार र परामर्श सेवाको बिस्तृत जानकारी							
१	२	३	४	५	६	७	८	९	१०	११	१२	१३	१४	१५	१६	१७	१८	१९	२०	२१	२२
								१	२									१	१. नियमित छ	१. सुधार छ	
																			२. नियमित छैन	२. सुधार छैन	
								१	२									१	१. नियमित छ	१. सुधार छ	
																			२. नियमित छैन	२. सुधार छैन	
								१	२									१	१. नियमित छ	१. सुधार छ	
																			२. नियमित छैन	२. सुधार छैन	
								१	२									१	१. नियमित छ	१. सुधार छ	
																			२. नियमित छैन	२. सुधार छैन	
								१	२									१	१. नियमित छ	१. सुधार छ	
																			२. नियमित छैन	२. सुधार छैन	
								१	२									१	१. नियमित छ	१. सुधार छ	
																			२. नियमित छैन	२. सुधार छैन	
								१	२									१	१. नियमित छ	१. सुधार छ	
																			२. नियमित छैन	२. सुधार छैन	

महल नं ४ को कोड: १- NCD (नसर्ने रोग), २- Mental Health (मानसिक स्वास्थ्य), ३- Injury (चोटपटक)

छपाई : आ.व २०[illegible]/८०

CS Scanned with CamScanner
