# Supplementary material for: Building linkages between private pharmacies and public facilities to improve diabetes and hypertension care in urban areas of Nepal: a protocol for implementation research
Source: Arch Public Health. 2025 Jun 19;83:160. doi: 10.1186/s13690-025-01586-4 (PMC12178029; doi:10.1186/s13690-025-01586-4)
Supplement: Supplementary file 4 — Supplementary Material 4 [file 13690_2025_1586_MOESM4_ESM.pdf]

Ref Slip No:

Referral Slip

Y	Y	Y	Y	M	M	D	D
2	0						

Client Name: ______________________________

Age: ☐☐

Sex: ☐ Male ☐ Female ☐ Others

	Systolic	Diastolic	
First Reading:	☐☐☐	☐☐☐	mmHg
Second Reading:	☐☐☐	☐☐☐	mmHg
Third Reading:	☐☐☐	☐☐☐	mmHg

Glucose Level: ☐☐☐ mg/dl

Type:
- ☐ Random Sugar
- ☐ Fasting Sugar
- ☐ Postprandial (PP)

Health Facility Referred to: ______________________

Contact No of Referred Health Facility : ______________

Referred by: Institution:

Contact Number:

Description of services provided and Return Information

Date

2	0	8					

Return Information sent to: ______________

Clients full name: ______________

Age ☐☐

Gender ☐ Female ☐ Male ☐ Others

Services provided:

Name of the return information provider:

Designation:

Name of health facility:
