# Supplementary material for: Building linkages between private pharmacies and public facilities to improve diabetes and hypertension care in urban areas of Nepal: a protocol for implementation research
Source: Arch Public Health. 2025 Jun 19;83:160. doi: 10.1186/s13690-025-01586-4 (PMC12178029; doi:10.1186/s13690-025-01586-4)
Supplement: Supplementary file 5 — Supplementary Material 5 [file 13690_2025_1586_MOESM5_ESM.pdf]

Table 1: Summary of methods, participants and the RE-AIM domain

Methods	Participants	Sample size/strategy	Time points	RE-AIM/purpose
Client follow-up surveys	Purposive Sample of clients to include both hypertensive and diabetic cases and diversify based on gender and age group	At least 20 clients per health facility and per pharmacy	2 data points in each timeline below:<br>Baseline<br>Month 6<br>Month 12<br>Month 18	R – proportion of urban poor out of sampled clients<br>E – self reported change in BP, D2, risk factors<br>A – differences in adoption by pharmacy and by staff within pharmacies<br>I- experience of care from pharmacy, referral, unintended consequences and costs<br>M – continuation in implementation
Interviews with health workers at pharmacy and public health facilities	All health workers at pharmacy and public health facilities within the intervention	11 health workers at pharmacy and 6 health workers of primary care facilities and 1 hospital staff	Baseline<br>Month 6<br>Month 12<br>Month 18	A – differences in adoption by pharmacy and by staff and public health facilities<br>I – facilitators, barriers, unintended consequences, costs<br>M – continuation in implementation
Routine HMIS/DHIS2 records	All 6-primary care facilities and one referral hospitals	All OPD cases of hypertension and diabetes	Continuous over 18 months	E effectiveness in reducing D2 and hypertension<br>M – maintenance in management of D2 and hypertension

Referral slips	All health workers at pharmacy within the intervention	All captured	Continuous over 1 year (or 18 months?)	A- adoption<br>I- implementation
Qualitative interviews	PMC Health division officials, Health care provider		Month 6<br>Month 12<br>Month 18	I- policy/guideline, supervision system<br>M- perspectives on sustainability
In-depth Interviews	NCD Clients	20 interviews selected purposively from client survey<br>5 follow up (not taking up the referral)	Month 6<br>Month 12<br>Month 18	A-adoption<br>E- effectiveness in reducing D2 and hypertension<br>I- experience of care from pharmacy, referral, unintended consequences and costs<br>M – continuation in implementation
In-depth Interviews	Health workers at pharmacy	10 interviews	During the implementation	I- facilitators, barriers, unintended consequences, costs
Case Studies	NCD clients referred	5 interviews	During the implementation	I- facilitators, barriers, experience of care from the referral hospital
