# Supplementary material for: Building linkages between private pharmacies and public facilities to improve diabetes and hypertension care in urban areas of Nepal: a protocol for implementation research
Source: Arch Public Health. 2025 Jun 19;83:160. doi: 10.1186/s13690-025-01586-4 (PMC12178029; doi:10.1186/s13690-025-01586-4)
Supplement: Supplementary file 7 — Supplementary Material 7 [file 13690_2025_1586_MOESM7_ESM.pdf]

At risk population for Diabetes

- Increasing age, especially after 45 years of age.
- If a mother, father or sibling has type 2 diabetes.
- Those with insufficient physical activity.
- Those who consume unhealthy foods such as high calorie, greasy, sugary and low fiber foods.
- Those who smoke tobacco and consume alcohol.
- Those with obesity because the body's cells to become insulin resistant due to excess fats in the body.
- Diabetes is more likely to occur to those individuals who have high blood pressure, stroke or high cholesterol level.
- If the pancreas gland doesn't work well or the body can't use insulin, it can lead to high blood sugar and a higher risk of diabetes.
- In some pregnant women, pregnancy causes hormonal changes and weight gain, which can make insulin less effective, leading to a higher risk of diabetes.

At risk population for Hypertension

- Those with unhealthy diet consumption practice (excessive use of salt, oily food)
- Those who are physical inactive and has obesity
- Those who smoke and use tobacco products
- Those who consume alcohol
- Those with anxiety/mental stress
- Increasing age
- Men have a higher risk of developing hypertension than women
- People of certain ethnicities have a higher risk of developing the disease
- Hereditary traits
