# Supplementary material for: Building linkages between private pharmacies and public facilities to improve diabetes and hypertension care in urban areas of Nepal: a protocol for implementation research
Source: Arch Public Health. 2025 Jun 19;83:160. doi: 10.1186/s13690-025-01586-4 (PMC12178029; doi:10.1186/s13690-025-01586-4)
Supplement: Supplementary file 8 — Supplementary Material 8 [file 13690_2025_1586_MOESM8_ESM.pdf]

Client Survey Questionnaire

Section 1: Demographic Characteristics

Questions	Responses	Code
Name of the respondent	……………………………………………………….	
Enter the ID of the respondent	……………………………………………………….	
Name of the health facility from where contact information was obtained	……………………………………………………….	
Select the type of health facility	Pharmacy<br>Health Post<br>Urban Health Center<br>Sishuwa Hospital	1<br>2<br>3<br>4
Age of the respondent	☐☐ (Completed years)	
Gender of the respondent	Male<br>Female<br>Others	1<br>2<br>96
Address of the respondent	……………………….<br>Ward Number	
Tole name	……………………………<br>Tole	
Marital status of the respondent	Married<br>Divorced<br>Separated<br>Widowed<br>Never- Married	0<br>1<br>2<br>3<br>4
What is your occupation? That is, what kind of work does you mainly do? (Source: NDHS, 2022)	Services<br>Unskilled Manual<br>Skilled Manual<br>Professional/ technical/ managerial	0<br>1<br>2<br>3

	Agriculture<br>Home maker<br>Unemployed<br>Other (Specify)…………………………….	4<br>5<br>6<br>96
What is the main source of income for your family? (Source: NDHS, 2022)	Daily wage labor<br>Skilled labor<br>Government<br>Non-government<br>Petty business<br>Business<br>Farmer (Agriculture/ Animal husbandry)<br>Pension<br>Self- employed<br>Foreign employment<br>Other (Specify)…………………………….	0<br>1<br>2<br>3<br>4<br>5<br>6<br>7<br>8<br>9<br>96

Section 2: Care seeking behavior

Questions	Options	Code
Do you have health insurance?	Yes<br>No	1<br>0
Currently, what health problems do you have?<br>(Select multiple if needed)	Hypertension<br>Diabetes Mellitus<br>None<br>Other (Specify)………	0<br>1<br>2<br>96
Do you currently take medication for hypertension?	Yes<br>No	1<br>0
If no, why not?	Side effect of medicines<br>Cost of medicines<br>Fear of being habitual<br>Lack of trust towards health provider<br>Need to consume lifelong<br>Control via lifestyle modification<br>Other (Specify)…………………………….	0<br>1<br>2<br>3<br>4<br>5<br>96
Where were you diagnosed with hypertension?		

	…………………………………………………<br>(Name/ Type of facility)	
How long ago were you diagnosed with hypertension?	………………………………………………….<br>(Days/ weeks/ months/ years)	
Do you currently take medication for diabetes?	Yes<br>No	1<br>0
If no, why not?	Side effect of medicines<br>Cost of medicines<br>Fear of being habitual<br>Lack of trust towards health provider<br>Need to consume lifelong<br>Control via lifestyle modification<br>Other (Specify)……………………………….	0<br>1<br>2<br>3<br>4<br>5<br>96
Where were you diagnosed with diabetes?	………………………………………………………….<br>(Name/ Type of facility)	
How long ago were you diagnosed with diabetes?	………………………………………………….<br>(Days/ weeks/ months/ years)	
Last time when you visited the ………………., what health problems related services did you get?	Hypertension<br>Diabetes Mellitus<br>Other (Specify)…………………………………..	1<br>2<br>96
What type of services did you receive from the ……………………………?	Prescription of medicines<br>Dispense<br>Counseling<br>Blood pressure checkup<br>Sugar check-up<br>Other (Specify)……………	0<br>1<br>2<br>3<br>4<br>96
Please ask the outcome of BP checkup or sugar checkup if they remember.	High blood pressure<br>Normal blood pressure<br>High sugar<br>Normal sugar<br>Can't recall<br>Other (Specify)……………………………….	1<br>2<br>3<br>4<br>99<br>96

Did the…………………asked you for follow up visit or do repeat measurement of blood pressure in same facility or other facilities?	Yes<br>No	1<br>0
If yes, in how many days were you asked to repeat measurement ?	…………………………………………..	
Why did you prefer ……………over other health facility such like health post, other urban health centre?<br>(Multiple Choice)	Proximity<br>Affordability<br>Referred by health care providers<br>Recommended by friends/ relatives<br>Prior (good) experience<br>Availability of medicines, equipment and services<br>Short waiting time<br>Good healthcare provider skills<br>Staff shows respect<br>Only facility available<br>Covered or assigned by insurance<br>Other (Specify)………………………………………	0<br>1<br>2<br>3<br>4<br>5<br>6<br>7<br>8<br>9<br>10<br>96
Since the last time you visited …………………, did you visit any other health facilities?	Yes<br>No	1<br>0
If yes, which facility did you visit ?	……………………………………………	

Section 3: Cost questionnaire

Household Characteristics	Options	Code
How much money do you spend on your last visit to the ………………………………?		
Consultation Fee/ Registration Charge (in rupees)	………………………………………rupees	
Diagnostic Test (in rupees)	………………………………………rupees	
Medicines for hypertension (in rupees)	………………………………………rupees	
For how many days were the medicines provided for?	……………………………………days	
Medicines for diabetes (in rupees)	………………………………………rupees	
For how many days were the medicines provided for?	……………………………………days	
Travel cost to ………………. (both way) [in NRs]	………………………………………rupees	
Do you have to skip your work to seek health care services from the health facility?	Yes<br>No	1<br>0
How much time was taken to visit to the ………….. during your last visit ? (sum both ways)	…………………………………….<br>(minutes/hour)	

How much waiting time was taken during your last visit to the……………..?	…………………………………….<br>(minutes/hour)	
How much time was taken to receive the services during your last visit at the ……………….?	…………………………………….<br>(minutes/hour)	
Did the visit reduce your per day earnings?	Yes<br>No<br>Don't know	1<br>0<br>98

Section 4: Risk Factors Questionnaire

Tobacco and Alcohol Consumption	Options	Code
Have you ever smoked cigarettes in lifetime?	Yes<br>No	1<br>0
In past 30 days, how often did you smoke cigarettes ?	Not at all<br>Somedays<br>Everyday	0<br>1<br>2
How many days a month?	……………………………………….days	
On the days you smoke, on average, how many cigarettes do you smoke each day?	……………………………………..<br>Number of cigarettes	
Do you currently smoke or use any other type of tobacco every day, some days, or not at all?	Not at all<br>Somedays<br>Everyday	0<br>1<br>2
What other type of tobacco do you currently smoke or use?	Pipes full of Tobacco/ Sulpha/ Chilum<br>Cigars<br>Water Pipe<br>Chewing Tobacco (Gutka/ Khaini)<br>Betel Quid with Tobacco<br>Vape<br>Bidi<br>Other (Specify)………………………………	0<br>1<br>2<br>3<br>4<br>5<br>6<br>96
Now I would like to ask you some questions about drinking alcohol.		
Have you ever consumed any alcohol, such as beer, wine, spirits, or local jaand, chyang etc.?	Yes<br>No	1<br>0

We count one drink of alcohol as one can or bottle of beer, one glass of wine, one shot of spirits, or one cup of jaand, chyang. During the last one month, on how many days did you have at least one drink of alcohol?	Did not have even one drink<br>Every Day/ Almost Every Day<br>Sometimes	0<br>1<br>2
Number of days in month	………………………….days	
In the last one month, on the days that you drank alcohol, how many drinks did you usually have per day?	……………………………….<br>Number of Drinks	
Did you receive any information on tobacco/ alcohol during you last visit to…………………………?	Yes<br>No	1<br>0
If yes, were all the messages clear for you to understand and act on?	Yes<br>No	1<br>0
**Physical Activity**		**Code**
How many times a week do you usually do 20 minutes of vigorous physical activity that makes you sweat or puff and pant? (for example, jogging, heavy lifting, digging, aerobics, or fast bicycling)	None<br>1-2 times/week<br>3 times/week<br>>3 times/week	0<br>1<br>2<br>3
How many times a week do you usually do 30 minutes of moderate physical activity or walking that increases your heart rate or makes you breathe harder than normal? (for example, mowing the lawn, carrying light loads, bicycling at a regular pace)	None<br>1-2 times/week<br>3- 5 times/week<br>>5 times/week	0<br>1<br>2<br>3
Were you provided any information on exercise/physical activity by the health facility? Prompt: verbal messages, IEC materials	Yes<br>No	1<br>0
If yes, were all the messages clear for you to understand and act on?	Yes<br>No	1<br>0
**Diet**		Code
What do you think that too much salt in your diet can do to your health? (Multiple response)	Nothing, more salt is good for health<br>Increase blood pressure<br>Kidney disease<br>Asthma<br>Cancer<br>Other (Specify)……………………..<br>Don't know	0<br>1<br>2<br>3<br>4<br>96<br>98
Do you do any of the following on a regular basis to control your salt intake?		
Avoid/ minimize consumption of processed foods such as achaar or papad	Yes<br>No	1<br>0
Reduced consumption of packaged food like instant noodles, chips in last one week compared to previous lifestyle	Yes<br>No	1<br>0

Use spices instead of salt when cooking	Yes<br>No	1<br>0
Eating foods prepared at home only	Yes<br>No	1<br>0
Eat meals adding extra salt at the table	Yes<br>No	1<br>0
Cook meals such as pulse, curry adding lesser salt	Yes<br>No	1<br>0
Were you provided any information on salt intake at ………………….?	Yes<br>No	1<br>0
If yes, were all the messages clear for you to understand?	Yes<br>No	1<br>0

Section 5: Counselling

Questionnaire	Options	Code
When you visited the ………………… last time, what information regarding tobacco use was provided?<br><br>(Please check section 4: Q300)	Harmful effects of smoking<br>Ways to control smoking<br>Social and environmental consequences<br>Economic consequences<br>Benefits of quitting smoking<br>No information was provided<br>Can't remember<br>Other (Specify)…………………………….	0<br>1<br>2<br>3<br>4<br>98<br>99<br>96
Have you been referred to any specific smoking cessation programs or specialists?	Yes<br>No	1<br>0
When you visited the ………………. last time, what information regarding alcohol use was provided?	Ways to stop drinking alcohols<br>Social and environmental consequences<br>Economic consequences<br>Benefits of quitting smoking<br>No information was provided<br>Can't remember<br>Other (Specify)…………………………….	0<br>1<br>2<br>3<br>98<br>99<br>96
When you visited the …………………. last time, what information regarding diet was provided?	Healthy diet<br>High sugar diet<br>High fat diet	0<br>1<br>2

	High salt diet<br>No information was provided<br>Can't remember<br>Other (Specify)……………………………	3<br>98<br>99<br>96
When you visited the ……………….. last time, what information regarding physical activity was provided?	Types of physical activity<br>Benefits of physical activity<br>At least 150 minutes of moderate- intensity physical activity per week (such as brisk walking, mopping, vacuuming)<br>No information was provided<br>Can't remember<br>Other (Specify)……………………………	0<br>1<br>2<br><br><br>98<br>99<br>96

Section 6: Patient Satisfaction Questionnaire

**Variables**	**Strongly Agree**	**Agree**	**Disagree**	**Strongly Disagree**
Now I would like to ask your satisfaction when receiving services from……………………………				
I was satisfied with the overall care provided by my pharmacist.	3	2	1	0
I would recommend that health facility to people I know.	3	2	1	0
If needed, I would continue seeing this health facility for my healthcare needs.	3	2	1	0
The overall care provided by the healthcare providers meet my expectations.	3	2	1	0

Section 7: Medication Adherence

**Questions**		**Code**
Do you sometimes forget to take your hypertension/ diabetes mellitus pills?	Yes<br>No	1<br>0
People sometimes miss taking their medications for reasons other than forgetting. Thinking over the past two weeks, were there any days when you did not take your hypertension/ diabetes mellitus medicine?	Yes<br>No	1<br>0
Have you ever cut back or stopped taking your medication without telling your doctor, because you felt worse when you took it?	Yes<br>No	1<br>0
When you travel or leave home, do you sometimes forget to bring along your hypertension/ diabetes mellitus medication?	Yes<br>No	1<br>0

Did you take your hypertension/ diabetes mellitus medicine yesterday?	Yes<br>No	1<br>0
When you feel like your hypertension/ diabetes mellitus is under control, do you sometimes stop taking your medicine?	Yes<br>No	1<br>0
Taking medication everyday is a real inconvenience for some people. Do you ever feel hassled about sticking to your blood pressure treatment plan?	Yes<br>No	1<br>0
How often do you have difficulty remembering to take all your medications? (Please circle the correct number)	Never/ Rarely<br>Once in a while<br>Sometimes<br>Usually<br>All the time	0<br>1<br>2<br>3<br>4

Section 8: Follow up and Referral Questionnaire

**Questions**		**Code**
Were you referred to another center from the…………………..?	Yes<br>No	1<br>0
If yes, where were you referred to?	……………………………………………………<br>(Name/ Type of health facility)	
Did you ask for the referral yourself?	Yes<br>No	1<br>0
Did you get the referral slip from ………………………health facility?	Yes<br>No	1<br>0
Did you go to the referred health facility after the referral?	Yes<br>No	1<br>0
If no, what is the reason behind not visiting the referred health facility?	Lack of perceived illness<br>Distance from home<br>Lack of guiding instructions/ reminder<br>Acute health conditions/ disability<br>Long waiting hours<br>Poor quality of medications<br>Convinced by others<br>No time for a visit<br>Other required medicines not available	0<br>1<br>2<br>3<br>4<br>5<br>6<br>7<br>8

	Others (Specify)……………………………….	96
If yes, what are the reasons for going to the referred health facility?	Information from ………….. health facility<br>Self-awareness<br>Specialized care<br>Others<br>(Specify)………………………………………..	0<br>1<br>2<br>96
Do you plan on going to the referred centre in the future?	Yes<br>No	1<br>0

Section 9: Validation of services

Questionnaire	Options	Code
In your last visit to ………………., how many times did the health professional check your blood pressure?	…………………………times	
In your last visit to …………………., did the health professional check your blood glucose level?	Yes<br>No	1<br>0
How many times ………………….measured blood glucose level ?	……………………………times	
Did the health professional in ……………….. measured your height?	Yes<br>No	1<br>0
Did the health professional in ……………….. measured your weight?	Yes<br>No	1<br>0
What information regarding the symptoms of diabetes was provided?	Excessive Thirst (Polyuria)<br>Frequent passage of urine (Polyuria)<br>Feeling hungry frequently (Polyphagia)<br>Dry mouth<br>Loss of weight<br>Painful/ burning urination<br>Feeling tired and weak<br>Tingling sensation in hand and feet<br>Blurred vision<br>Didn't give any information<br>Other (Specify)………………………………………….<br>Can't Remember	0<br>1<br>2<br>3<br>4<br>5<br>6<br>7<br>8<br>98<br>96<br>99

What information regarding the symptoms of hypertension was provided at ……………………?	Nausea<br>Vomiting<br>Headache<br>Vertigo<br>Epistaxis (nose bleeds)<br>Swelling of hand and feet<br>Difficulty breathing/ Shortness of breath<br>Blurred Vision<br>Didn't give any information<br>Other (Specify)……………………………………<br>Can't Remember	0<br>1<br>2<br>3<br>4<br>5<br>6<br>7<br>98<br>96<br>99
What information regarding the risk factors of diabetes was provided at …………………..?	Unhealthy Diet<br>Lack of physical activity/ Obesity<br>Obesity<br>Smoking of tobacco and use of smokeless tobacco products<br>Alcoholism<br>Growing age<br>Hereditary<br>History of insulin resistance, strokes or hypertension<br>Didn't give any information<br>Other (Specify)……………………………………….<br>Can't Remember	0<br>1<br>2<br>3<br>4<br>5<br>6<br>7<br>98<br>96<br>99
What information regarding the risk factors of hypertension was provided at …………………..?	Unhealthy Diet<br>Lack of physical activity<br>Obesity<br>Smoking of tobacco and use of smokeless tobacco products<br>Alcoholism<br>Stress<br>Growing age<br>Gender<br>Hereditary<br>Didn't give any information<br>Other (Specify)……………………………………….<br>Can't Remember	0<br>1<br>2<br>3<br>4<br>5<br>6<br>7<br>8<br>98<br>96<br>99
What information regarding the complication of diabetes was provided at …………………..?	Stroke<br>Retinopathy	0<br>1

	Cardiomyopathy<br>Proteinuria<br>Neuropathy<br>Diabetic foot<br>Didn't give any information<br>Others<br>Can't Remember	2<br>3<br>4<br>5<br>98<br>96<br>99
What information regarding the complication of hypertension was provided at…………………?	Renal failure/ chronic kidney disease<br>Heart attack/ left ventricular hypertrophy/ heart failure<br>Epistaxis<br>Stroke<br>Hypertensive Retinopathy<br>Swelling of feet<br>Aneurysm Bleeding<br>Ascites<br>Didn't give any information<br>Others<br>Can't Remember	0<br>1<br>2<br>3<br>4<br>5<br>6<br>7<br>98<br>96<br>99
Did the health professional in ……………………provided you instruction about proper ways of consuming the medicine?	Yes<br>No	1<br>0
Were you provided with any brochure related to health information at ………………….?	Yes<br>No	1<br>0
Were the information provided in the brochure easy for you to understand?	Yes<br>No	1<br>0
Do you think you will be able to follow the advice provided at……………………?	Yes<br>No	1<br>0
If not, which advice is most challenging for you to follow?	Tobacco cessation<br>Avoiding alcohol<br>Doing physical activity as recommended<br>Avoiding salt consumption as recommended<br>Adhering to the prescribed medicines<br>Other (Specify)……………………………………….	0<br>1<br>2<br>3<br>4<br>96
How can health information delivery be improved at the health facility?<br>Prompt: If brochure, leaflets are helpful, get their opinion on how it should look	………………………………………………………………..	

Section 10: Socio economic Characteristics

Household Characteristics	Options	Code
How many people are there in your household including yourself?	…………………………<br>Number of people	
How many rooms that are used as bedrooms are there in your household ?	…………………………..<br>Rooms	
Do you have a room available to rent to others?	Yes<br>No	1<br>0
What is dwelling roof made up of?	Zinc/tin/chidar<br>Cement Slope<br>Khar/Paral/Real straw<br>Tile/Khapada/Jhingati<br>Stone/Slate<br>Wood/Wood planks<br>Soil<br>Other (Specify)……………………………	0<br>1<br>2<br>3<br>4<br>5<br>6<br>96
What are the walls of dwelling made up of?	Brick and stone with soil joints<br>Brick and stone with cement joints<br>Wood/wood planks<br>Bamboo materials<br>Raw/unfired brick<br>Zinc/tin<br>Prefab<br>Block<br>Other (Specify)…………………………..	1<br>2<br>3<br>4<br>5<br>6<br>7<br>8<br>96
What is the main source of drinking water for members of your households?	Piped into dwelling<br>Piped to yard/ plot<br>Piped to neighbour<br>Public tap/ Standpipe<br>Tube well or Borehole<br>Protected well<br>Unprotected well<br>Protected Spring<br>Unprotected Spring	0<br>1<br>2<br>3<br>4<br>5<br>6<br>7<br>8

	Rainwater<br>Tanker Truck<br>Cart with small tank<br>Surface Water (River/ Dam/ Lake/ Pond/ Stream/ Canal/ Irrigation Channel)<br>Bottled Water<br>Other (Specify)……………………………….	9<br>10<br>11<br>12<br><br>13<br>96
What kind of toilet facility do members of your household usually use?<br>(Ask permission to observe the facility)	Flush Toilet (septic tank)<br>Flush Toilet (public sewerage)<br>Pit Latrine<br>Public Toilet<br>No Toilet Facility<br>Others (Specify)…………………	1<br>2<br>3<br>4<br>5<br>96
Do you share this toilet facility with other households?	Yes<br>No	1<br>2
If yes, where is this toilet facility located?	In own dwelling<br>In own yard/ plot<br>Elsewhere	1<br>2<br>3
Does your household have a handwashing facility?	Yes<br>No	1<br>0
Does your household have water in the hand-washing place?	Yes<br>No	1<br>0
Availability of soap, liquid, etc<br>(Ask permission to observe the facility)	Yes<br>No	1<br>0
Does this household have a television?	Yes<br>No	1<br>0
Does the household have it's own business?	Yes<br>No	1<br>0
If yes, is the business registered?	Yes<br>No	1<br>0
Do you own a agricultural land in Pokhara?	Yes<br>No	1<br>0
Have you migrated to this area for reasons of economic necessity?	Yes<br>No	1<br>0
If yes, how many years ago did you migrate?	……………………..years	

What is the highest academic qualification among the household members?	No formal education<br>Lower basic education (1-5)<br>Upper basic education (6-8)<br>Lower secondary (9-10)<br>Higher secondary (11-12)<br>Bachelor’s degree<br>Master's Degree or above	0<br>1<br>2<br>3<br>4<br>5<br>6
What is the academic qualification of the household head?	No formal education<br>Lower basic education (1-5)<br>Upper basic education (6-8)<br>Lower secondary (9-10)<br>Higher secondary (11-12)<br>Bachelor’s degree<br>Master's Degree or above	0<br>1<br>2<br>3<br>4<br>5<br>6
What is the occupation of the household head?	Services<br>Unskilled Manual<br>Skilled Manual<br>Professional/technical/managerial<br>Agriculture<br>Homemaker<br>Unemployed<br>Other (Specify)……………………………	0<br>1<br>2<br>3<br>4<br>5<br>6<br>96
How would you rate your neighbours overall in terms of financial and economic?	……………………………………………………	
How would you rate your household compared to your neighbours? (In terms of income and property)	………………………………………………………	

Section 11: Anthropometric Measurements

Anthropometric Measurements	Measurements	Code
First Systolic reading	………………………………. mm of Hg	
Frist Diastolic reading	………………………………. mm of Hg	
Second Systolic reading	………………………………. mm of Hg	

Second Diastolic reading	………………………………. mm of Hg	
Third Systolic reading	………………………………. mm of Hg	
Third Diastolic reading	………………………………. mm of Hg	
Height	……………………………….m	
Weight	………………………………. kg	
BMI	………………………………. Kg/ m^2	
During the past 8 hours have you had anything to eat or drink, other than water?	Yes<br>No	1<br>0
Blood Glucose level	…………………………………..mg/dl	

Client Survey Questionnaire (Follow Up)

Section 1: Demographic Characteristics

Questions	Responses	Code
Name of the respondent	……………………………………………………………	
Age of the respondent	☐☐ (Completed years)	
Gender of the respondent	Male<br>Female<br>Others	1<br>2<br>96
Address of the respondent	……………………….<br>Ward Number	
Tole name	……………………………<br>Tole	

Section 2: Care seeking behavior

Questions	Options	Code
Do you have health insurance?	Yes<br>No	1<br>0
Currently, what health problems do you have?<br>(Select multiple if needed)	Hypertension<br>Diabetes Mellitus<br>None<br>Other (Specify)………	0<br>1<br>2<br>96
Do you currently take medication for hypertension?	Yes<br>No	1<br>0

If no, why not?	Side effect of medicines<br>Cost of medicines<br>Fear of being habitual<br>Lack of trust towards health provider<br>Need to consume lifelong<br>Control via lifestyle modification<br>Other (Specify)……………………………….	0<br>1<br>2<br>3<br>4<br>5<br>96
Where were you diagnosed with hypertension?	……………………………………………………<br>(Name/ Type of facility)	
How long ago were you diagnosed with hypertension?	…………………………………………………<br>(Days/ weeks/ months/ years)	
Do you currently take medication for diabetes?	Yes<br>No	1<br>0
If no, why not?	Side effect of medicines<br>Cost of medicines<br>Fear of being habitual<br>Lack of trust towards health provider<br>Need to consume lifelong<br>Control via lifestyle modification<br>Other (Specify)……………………………….	0<br>1<br>2<br>3<br>4<br>5<br>96
Where were you diagnosed with diabetes?	……………………………………………………….	
How long ago were you diagnosed with diabetes?	…………………………………………………<br>(Days/ weeks/ months/ years)	

Section 3: Follow up and Referral Questionnaire

Questions		Code
Were you referred to another center from the…………………..?	Yes<br>No	1<br>0
If yes, name of the facility		

	………………………………………………….<br>(Name/ Type of health facility)	
Did you ask for the referral yourself?	Yes<br>No	1<br>0
Did you get the referral slip from ……………………….health facility?	Yes<br>No	1<br>0
Did you go to the referred health facility after the referral?	Yes<br>No	1<br>0
If no, what is the reason behind not visiting the referred health facility?	Lack of perceived illness<br>Distance from home<br>Lack of guiding instructions/ reminder<br>Acute health conditions/ disability<br>Long waiting hours<br>Poor quality of medications<br>Convinced by others<br>No time for a visit<br>Other required medicines not available<br>Others (Specify)…………………………….	0<br>1<br>2<br>3<br>4<br>5<br>6<br>7<br>8<br>96
If yes, what are the reasons for going to the referred health facility?	Information from …………. health facility<br>Information from HERD International field researchers<br>Self-awareness<br>Specialized care<br>Others (Specify)……………………………………..	0<br>1<br>2<br>3<br>96
Do you plan on going to the referred centre in the future?	Yes<br>No	1<br>0
When you visited the…………………..health facility, what health problems related services did you get?	Hypertension<br>Diabetes Mellitus<br>Others (Specify)………………………………..	1<br>2<br>96
What type of services did you receive from the ……………………………?	Prescription of medicines<br>Dispense<br>Counseling<br>Blood pressure checkup<br>Sugar check-up<br>Other (Specify)……………	0<br>1<br>2<br>3<br>4<br>96
Please ask the outcome of BP checkup or sugar checkup if they remember.	High blood pressure<br>Normal blood pressure	1<br>2

	High sugar<br>Normal sugar<br>Can't recall<br>Others (Specify)……………………………….	3<br>4<br>99<br>96
Since the last time you visited …………………, did you visit any other health facilities?	Yes<br>No	1<br>0
If yes, which facility did you visit ?	……………………………………………	
If yes, what are the reasons for going to the referred health facility?	Information from HERD International field researchers<br>Self-awareness<br>Specialized care<br>Others (Specify)………………………………………..	0<br>1<br>2<br>96
When you visited the…………………..health facility, what health problems related services did you get?	Hypertension<br>Diabetes Mellitus<br>Others (Specify)………………………………..	1<br>2<br>96
What type of services did you receive from the ……………………………?	Prescription of medicines<br>Dispense<br>Counseling<br>Blood pressure checkup<br>Sugar check-up<br>Other (Specify)……………	0<br>1<br>2<br>3<br>4<br>96
Please ask the outcome of BP checkup or sugar checkup if they remember.	High blood pressure<br>Normal blood pressure<br>High sugar<br>Normal sugar<br>Can't recall<br>Others (Specify)……………………………….	1<br>2<br>3<br>4<br>99<br>96

Section 4: Risk Factors Questionnaire

Tobacco and Alcohol Consumption	Options	Code
Have you ever smoked cigarettes in lifetime?	Yes<br>No	1<br>0
In past 15 days, how often did you smoke cigarettes ?	Not at all<br>Somedays<br>Everyday	0<br>1<br>2

How many days in 15 days?	……………………………………….days	
On the days you smoke, on average, how many cigarettes do you smoke each day?	…………………………………..<br>Number of cigarettes	
Do you currently smoke or use any other type of tobacco every day, some days, or not at all?	Not at all<br>Somedays<br>Everyday	0<br>1<br>2
What other type of tobacco do you currently smoke or use?	Pipes full of Tobacco/ Sulpha/ Chilum<br>Cigars<br>Water Pipe<br>Chewing Tobacco (Gutka/ Khaini)<br>Betel Quid with Tobacco<br>Vape<br>Bidi<br>Other (Specify)………………………………	0<br>1<br>2<br>3<br>4<br>5<br>6<br>96
Now I would like to ask you some questions about drinking alcohol.		
Have you ever consumed any alcohol, such as beer, wine, spirits, or local jaand, chyang etc.?	Yes<br>No	1<br>0
During the last 15 days, on how many days did you have at least one drink of alcohol?	Did not have even one drink<br>Every Day/ Almost Every Day<br>Sometimes	0<br>1<br>2
Number of days in the last 15 days	…………………………days	
In the last one month, on the days that you drank alcohol, how many drinks did you usually have per day?	………………………………<br>Number of Drinks	
**Physical Activity**		**Code**
How many times a week do you usually do 20 minutes of vigorous physical activity that makes you sweat or puff and pant? (for example, jogging, heavy lifting, digging, aerobics, or fast bicycling)	None<br>1-2 times/week<br>3 times/week<br>>3 times/week	0<br>1<br>2<br>3
How many times a week do you usually do 30 minutes of moderate physical activity or walking that increases your heart rate or makes you breathe harder than normal? (for example, mowing the lawn, carrying light loads, bicycling at a regular pace)	None<br>1-2 times/week<br>3- 5 times/week<br>>5 times/week	0<br>1<br>2<br>3
Diet		Code

What do you think that too much salt in your diet can do to your health? (Multiple response)	Nothing, more salt is good for health<br>Increase blood pressure<br>Kidney disease<br>Asthma<br>Cancer<br>Other (Specify)………………………<br>Don't know	0<br>1<br>2<br>3<br>4<br>96<br>98
Do you do any of the following on a regular basis to control your salt intake?		
Avoid/ minimize consumption of processed foods such as achaar or papad	Yes<br>No	1<br>0
Reduced consumption of packaged food like instant noodles, chips in last one week compared to previous lifestyle	Yes<br>No	1<br>0
Use spices instead of salt when cooking	Yes<br>No	1<br>0
Eating foods prepared at home only	Yes<br>No	1<br>0
Eat meals adding extra salt at the table	Yes<br>No	1<br>0
Cook meals such as pulse, curry adding lesser salt	Yes<br>No	1<br>0

Section 5: Medication Adherence

Questions		Code
Do you sometimes forget to take your hypertension/ diabetes mellitus pills?	Yes<br>No	1<br>0
People sometimes miss taking their medications for reasons other than forgetting. Thinking over the past two weeks, were there any days when you did not take your hypertension/ diabetes mellitus medicine?	Yes<br>No	1<br>0
Have you ever cut back or stopped taking your medication without telling your doctor, because you felt worse when you took it?	Yes<br>No	1<br>0
When you travel or leave home, do you sometimes forget to bring along your hypertension/ diabetes mellitus medication?	Yes<br>No	1<br>0
Did you take your hypertension/ diabetes mellitus medicine yesterday?	Yes<br>No	1<br>0

When you feel like your hypertension/ diabetes mellitus is under control, do you sometimes stop taking your medicine?	Yes<br>No	1<br>0
Taking medication everyday is a real inconvenience for some people. Do you ever feel hassled about sticking to your blood pressure treatment plan?	Yes<br>No	1<br>0
How often do you have difficulty remembering to take all your medications? (Please circle the correct number)	Never/ Rarely<br>Once in a while<br>Sometimes<br>Usually<br>All the time	0<br>1<br>2<br>3<br>4

Section 6: Anthropometric Measurements

Anthropometric Measurements	Measurements	Code
First Systolic reading	………………………………. mm of Hg	
Frist Diastolic reading	………………………………. mm of Hg	
Second Systolic reading	………………………………. mm of Hg	
Second Diastolic reading	………………………………. mm of Hg	
Third Systolic reading	………………………………. mm of Hg	
Third Diastolic reading	………………………………. mm of Hg	
Height	……………………………….m	
Weight	………………………………. kg	
BMI	………………………………. Kg/ m^2	
During the past 8 hours have you had anything to eat or drink, other than water?	Yes<br>No	1<br>0
Blood Glucose level	………………………………….mg/dl	
